# Association between Physical Activity and Sport Participation on Hemoglobin A1c Among Children and Adolescents with Type 1 Diabetes

**DOI:** 10.3390/ijerph18147490

**Published:** 2021-07-14

**Authors:** Kristi M. King, Jason R. Jaggers, Lindsay J. Della, Timothy McKay, Sara Watson, Amy E. Kozerski, Kimberly R. Hartson, Kupper A. Wintergerst

**Affiliations:** 1 Wendy Novak Diabetes Center, Division of Pediatric Endocrinology, School of Medicine, University of Louisville, Louisville, KY 40202, USA; Jason.Jaggers@louisville.edu (J.R.J.); timothy.mckay@louisville.edu (T.M.); sara.watson@louisville.edu (S.W.); kupper.wintergerst@louisville.edu (K.A.W.); 2 Department of Health and Sport Sciences, University of Louisville, Louisville, KY 40292, USA; 3 Department of Communication, University of Louisville, Louisville, KY 40292, USA; Lindsay.Della@louisville.edu; 4 Epidemiology and Cancer Control, St. Jude Children’s Research Hospital, Memphis, TN 38105, USA; amy.kozerski@stjude.org; 5 School of Nursing, University of Louisville, Louisville, KY 40292, USA; Kimberly.rapp@louisville.edu

**Keywords:** physical activity, pediatric, clinical exercise, sport medicine, diabetes

## Abstract

Purpose: To determine associations between physical activity (PA) and sport participation on HbA1c levels in children with type 1 diabetes (T1D). Method: Pediatric patients with T1D were invited to complete a PA and sport participation survey. Data were linked to their medical records for demographic characteristics, diabetes treatment and monitoring plans, and HbA1c levels. Results: Participants consisted of 71 females and 81 males, were 13 ± 3 years old with an average HbA1c level of 8.75 ± 1.81. Children accumulating 60 min of activity 3 days or more a week had significantly lower HbA1c compared to those who accumulated less than 3 days (*p* < 0.01) of 60 min of activity. However, there was no significant difference in HbA1c values based on sport participation groups. A multiple linear regression model indicated that PA, race, age, duration of diagnosis, and CGM use all significantly predicted HbA1c (*p* < 0.05). Conclusion: This study demonstrated the significant relationship between daily PA and HbA1c. Those in this sample presented with lower HbA1c values even if accumulating less than the recommended number of days of activity. Further, it was shown that sport participation alone may not be adequate enough to impact HbA1c in a similar manner.

## 1. Introduction

Children with type 1 diabetes (T1D) should engage in a minimum of 60 min of moderate to vigorous intensity physical activity (PA) per day, the same as children without T1D. However, due to the inability of the body’s natural response to control glucose fluctuations, care must be taken to prevent incidents of low blood glucose levels (hypoglycemia) or high blood glucose levels (hyperglycemia) during and after physical activity [1,2,3,4,5,6]. A continuous, dynamic, and complex balance of insulin administration, nutrition, PA, and monitoring of blood glucose levels are required to manage T1D. Children with T1D can still maintain a healthy and physically active lifestyle through recreational and general play as well as by participating in sports and organized activities as long as necessary safety precautions are taken. Scientific guidance exists regarding glucose targets for safe and effective participation in PA as well as nutritional and insulin dose adjustments to protect against PA-related glucose excursions [7,8,9].

There have been a number of studies investigating the impacts of sedentary lifestyles compared to physically active lifestyles in patients with T1D outcomes [10,11,12,13], however the majority of studies were conducted with older populations and there is limited information in regards to PA and sports participation available for children with T1D. Participation in sports is an excellent way for children to accumulate PA, while also gaining valuable social and life skills. Furthermore, studies have shown that children who participate in sports were more likely to meet PA guidelines than children who do not participate in sports [14].

There is evidence to suggest that PA in youth with T1D can contribute to decreases in HbA1c [10,11,12,13]. For example, among children with T1D, less active children have been known to exhibit poor glycemic control and significantly higher HbA1c levels compared to children who accumulate more physical activity most, if not all, days of the week [15,16]. Although sport participation can be a way for T1D patients to be physically active and perhaps improve HbA1c, no research studies have analyzed the differences in HbA1c levels and sport participation in children with T1D. Therefore, the purpose of this study was to examine the associations between PA and sport participation on HbA1c levels in children with T1D. The influence of sociodemographic characteristics and use of diabetes management tools (e.g., insulin pump) on HbA1c were also explored.

Identifying more direct benefits of increased PA on diabetes management and glucose control may aid diabetes care teams in developing individualized prescriptions to increase daily PA in a safe manner due to their understanding of acute and chronic physiological response for children managing T1D. The most prevalent and accessible measure in determining glucose control is the hemoglobin A1c (HbA1c) test which is an indicator of the average blood glucose levels over the past 3 months. Children managing T1D should strive for HbA1c levels less than 7% as an elevated HbA1c level is known to increase the risk for diabetes related complications [17]. It is important for health professionals, parents, and even teachers to understand children’s PA and sport participation behaviors and how they are associated with glucose control in children with T1D.

## 2. Materials and Methods

### 2.1. Procedures

This cross-sectional study was conducted at the Wendy Novak Diabetes Center, a nationally certified pediatric diabetes care and academic medical center located in the Southeastern United States. The study was approved by the University Institutional Review Board (IRB #18.0713) and parental/guardian informed consent and child assent were obtained prior to study participation. Patients between the ages of 7 to 17 years old with T1D were invited to participate in this study while in the diabetes care clinic for their regularly scheduled clinic appointment. Interested participants were given an iPad to complete the informed consent/assent process and the PA and sport participation survey which took approximately 10 min to complete. The survey was housed within REDCap [18] on a secure server. Survey data were linked to the clinical database at the Wendy Novak Diabetes Center by the researchers utilizing the patients’ medical record numbers to retrieve the measured HbA1c values from that same day appointment for each participant. Once data were collected and merged, the full dataset was de-identified for analysis.

### 2.2. Demographic Characteristics, Diabetes Monitoring, Treatment Plans and Outcomes

Demographic characteristics utilized were participants’ age, duration of T1D diagnosis, ethnicity, race, gender, insurance type, and body composition. Instead of using the more traditional body mass index (BMI) Z score, this study reports children’s body composition using the tri-ponderal mass index (TMI) calculation of kg/m^3^ as it has been shown to provide better reliability in determining the body composition of growing children [19,20]. Diabetes monitoring was assessed whether a participant used a continuous glucose monitor (CGM), an insulin pump, or relied solely on self-injections and monitoring. The dependent outcome variable HbA1c level, a continuous variable ranging from 6.4 to 14.9%, was obtained from that day’s clinical lab measures.

### 2.3. Physical Activity and Sport Participation

Two survey items from the 2017 Youth Risk Behavior Surveillance System (YRBSS) questionnaire were used in this study [21]. PA participation was assessed by the following item: “During the past 7 days, on how many days were you physically active for a total of at least 60 min per day? (Add up all the time you spend in any kind of physical activity that increased your heart rate and made you breathe hard some of the time)” with response options ranging from 0–7 days. Sport participation was assessed by the following item: “During the past 12 months, on how many sports teams did you play? (Include any teams run by your school or community)” with response items ranging from 0 to 3 or more teams. Both YRBSS items have been shown to be valid and reliable in populations of children of similar ages [22].

### 2.4. Data Analysis

Survey data and clinical data were imported from REDCap into a spreadsheet for analysis using statistical software SPSS (IBM SPSS Statistics, Version 25.0. Armonk, NY, USA). All variables were tested for normality in which it was discovered that the HbA1c measure did not follow a normal distribution. Therefore, HbA1c was log transformed prior to statistical analysis. PA and sport participation were analyzed individually by separating participants into groups according to number of days per week of PA and sport participation. This was done in an effort to further examine the differences between self-reported weekly PA, sport participation, and HbA1c. For the first analysis participants were grouped into tertiles according to number of days they reported to have accumulated 60 min or more of PA within the past week. Levels of PA were determined by splitting the data into three equal groups, which are as follows: Tertile 1: 0–2 days/week of ≥60 min/PA; Tertile 2: 3–4 days/week of ≥60 min/PA; Tertile 3: 5–7 days/week of ≥60 min/PA. To test the dependent variable HbA1c a one-way ANOVA was used to determine significant differences between groups. Post hoc between-groups comparisons were carried out using Tukey’s HSD to account for multiple testing. Due to the unequal distribution of sample sizes for race and ethnicity a Kruskall-Wallis test was used for differences in those groups. For sport participation, participants were separated into two groups based on whether or not they participated in an organized sport within the past year. Since this placed the sample into unevenly distributed groups, the non-parametric independent-samples Mann-Whitney U test was used for analysis.

In the secondary analyses, multiple linear regression models with HbA1c as the dependent variable were employed to examine the association between HbA1c and PA. To examine the relationships between PA and other known covariates on HbA1c independent of one another two multiple regression analyses were run with PA as the independent variable and HbA1c as the dependent variable in all models. The first model adjusted for potential confounding factors that were identified as being significantly associated to HbA1c through a Pearson’s correlation analysis. A second regression model also controlled for the independent variables of the other model while including additional sociodemographic and diabetes management variables. To allow for direct comparison across covariates, results of the linear regression analysis also present the standardized beta coefficient. A *p*-value of < 0.05 was considered statistically significant for all statistical analyses.

## 3. Results

### 3.1. Characteristics of the Participants

A total of 153 participants submitted a completed survey. One outlier was identified and removed from the dataset leaving a total of 152 participants in the final analysis. Table 1 shows the sample characteristics and variables of all participants, as well as the separation into groups according to daily PA. Participants consisted of 71 females (46.71%) and 81 males (53.29%), were 13 ± 3 years of age with an average HbA1c level of 8.75 ± 1.81. They were, on average, physically active for 60 min or more 3.49 ± 1.95 days per week. Only 7.9% (*n* = 12) met the minimal recommendation of daily PA although almost two-thirds played sports (*n* = 98, 64.1%).

### 3.2. Associations between Frequency of Physical Activity, Sport Participation and Diabetes Health Measures

**Figure 1:**
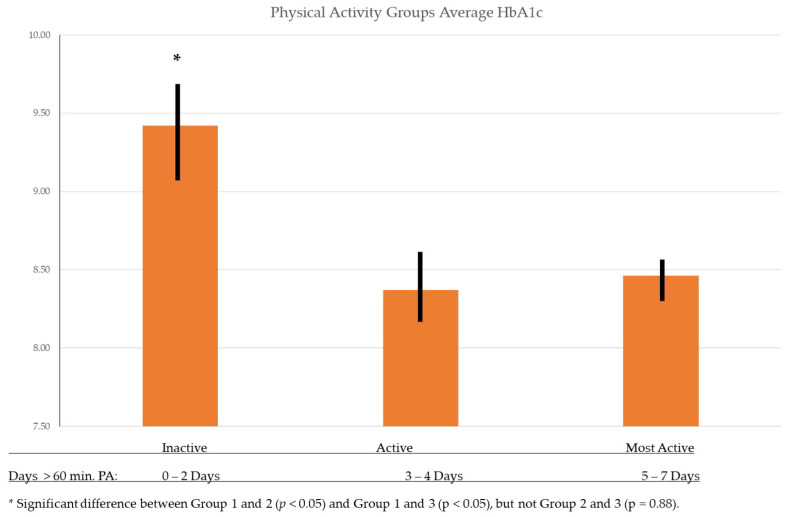
Differences in HbA1c Across Physical Activity Groups.

A one-way ANOVA showed statistically significant differences between PA groups indicating lower values of HbA1c as daily PA increased (*p* < 0.01) (Table 1). In a Tukey post-hoc analysis of the sub-groups it was further shown that the significant differences were observed between the Inactive and Active groups in terms of HbA1c (*p* = 0.01), as well as the Inactive and Most Active groups (*p* = 0.03) (Figure 1). Comparisons between PA groups Active and Most Active did not have a significant difference in HbA1c (*p* = 0.88). To examine the association between sport participation and HbA1c, an independent-samples Mann-Whitney U test was used and revealed no significant difference between sport participation and HbA1c levels (*p* = 0.27).

Pearson’s correlation revealed significant relationships were also observed between HbA1c and age (*r* = 0.19, *p* = 0.01), race (*r* = −0.18, *p* = 0.03), CGM use (*r* = −0.22, *p* = 0.006), and duration of T1D diagnosis (*r* = 0.23, *p* = 0.004). In order to analyze factors influencing HbA1c, multiple regression analyses were performed in the whole population with total days of PA as the independent variable instead of the respective PA groups. Results from the regression analyses indicated that the more days a child engages in 60 min or more of PA the lower their HbA1c compared to less active children from this sample (Table 2). The first model adjusted for variables that had a significant correlation with HbA1c (i.e., disease duration, CGM use, age, and race), in which it was found that in addition to daily PA, other significant predictors of HbA1c included race, CGM use, and disease duration, but not age. After adjustment for all sociodemographic variables (Model 2), the association with daily PA was still statistically significant with a β-coefficient that changed to −0.18 (95% CI: −0.32 to −0.02) indicating an even stronger relationship when taking into consideration other impacts on health like insurance and common medical devices designed to help improve diabetes management as indicated in these models (i.e., CGM use and duration of diagnosis).

## 4. Discussion

This study sought to determine the extent to which PA and sport participation are associated with HbA1c levels in children with T1D as well as explore the strength of relationships among demographics, diabetes treatment, HbA1c, PA, and sport participation characteristics. The results of this study indicated that children’s HbA1c improved with PA, but that sport participation alone may not be enough activity to have any positive impact on HbA1c according to this sample. When separated by days of PA, results showed that children who accumulated 60 min of PA at least 3 days or more out of the week presented with lower HbA1c levels when compared to children achieving 60 min of PA only two days out of the week or less. Compared to the Inactive PA group this significant finding was also observed in children who only accumulated 3 to 4 days of PA, which is less than the current recommended amount of 60 min or more of moderate to vigorous physical activity every day of the week (Figure 1). This trend would suggest potential benefit for those children who struggle to meet the current guidelines every single day of the week.

Although almost 2/3 of the children reported playing 1 or more sports in the previous year, they were only physically active for at least 1 h or more on average 3.49 days per week. Of important practical and clinical consideration is that less than 8% of the children in this sample met the recommended duration of one hour and frequency of 7 days per week of PA. Considering that the American Diabetes Association recommends PA as a key behavior in managing T1D effectively, the children in this study were quite sedentary. Results from 2017 YRBSS data indicated that nationally 26.1% (17.5% girls and 35.3% boys) and state-wide 22.0% (12.8% girls and 31.0% boys) were physically active [21]. The sample of children in this study fell far from meeting these PA minimal guidelines.

Sport participation has long been touted as a way for children to increase duration in PA and improve overall health and T1D management [23]. Specifically, previous research has shown PA affected HbA1c levels: the more days active, the lower the child’s HbA1c level. Beraki and colleagues [15] found that less active children had an average HbA1c level of 8.8 ± 1.5, while more active children had an average HbA1c level of 7.7, SD ± 1.0. Thus, sport participation, at first glance, appears to be a promising avenue for children with T1D to consider when trying to increase their PA levels. Interestingly 64.4% of the children in this sample played on at least 1 sports team in the past year, more than the national results 54.3% (49.3% girls and 59.7% boys) and statewide results of 48.3% (46.5% girls and 50.5% boys). The findings from this study suggest that daily PA had more of an impact on reducing HbA1c levels for children with T1D than sport participation alone. These findings would also suggest that children participating in sports is not enough activity by itself to meet current recommended guidelines.

A systematic review of 23 studies with meta-analysis indicated that PA is important for diabetes management but there is a lack of studies promoting sustained PA [24]. There remains a lack of knowledge of how to safely support and promote PA in people with T1D. Results from research on self-management of T1D in the school setting has identified that while some schools have procedures that support the participation of youth with T1D in sports, other schools require that the parents be present in order for their child to participate. A lack of knowledge of T1D among school staff and coaches can be a barrier to PA among youth with T1D [25,26].

Further, results from the exploration into the demographic and PA relationship indicate that there was a negative relationship between physical activity and age, meaning that older children were less active which is concurrent in the research. Recent YRBSS findings indicate that only 35% of high school boys and 18% of high school girls engaged in 60 min of daily PA. Younger children are typically more active than older children. For example, children 6–11 years engaged in 88 min of daily physical activity compared to adolescents aged 12–15 years (33 min), and 16–19 years (26 min) [27].

Children’s participation in physical activity may be limited due to lack of access to medically supervised PA opportunities for children managing T1D, and further complicated by logistical or financial reasons, especially among minority or low socioeconomically disadvantaged communities [28]. The participants’ demographic characteristics of race, ethnicity, and gender were an approximate reflection of the population in the state and surrounding geographical areas. It is interesting to note that most of the children were enrolled in Medicare/Medicaid which can be indicative of lower socioeconomic status, especially considering that on average, a family with a child with T1D pays extra in medical care coverage, insurance, and expenses per year than a family without T1D. A little over one-half of the sample had additional private insurance and about one-half of the sample used a CGM as well. What is most concerning with the results of the current study is the observation of other significant predictors of HbA1c including race and CGM use which would provide additional evidence in support of racial disparities that exist in healthcare. Even with a smaller sample size, it was found that participants who identified as Black or Other had a significantly higher HbA1c compared to Caucasian participants (10.46 vs. 8.52; *p* < 0.001) after running a separate non-parametric independent samples test.

Although the results from this study did not detect statistical significance in HbA1c outcomes and sport participation, further research is encouraged from the clinical and exercise communities. With strong evidence indicating the importance of daily PA and sport participation on health, proper growth, and motor function for children of all ages recent initiatives have been put in place to help children and youth increase daily PA through sport participation [29]. Thus, hypothetically sport participation can be a way for T1D patients to improve HbA1c, yet more research studies are required to specifically analyze the differences in HbA1c levels and sport participation in children with T1D. For children with T1D, the diligence required to adequately maintain blood glucose levels, while still participating in sports, can be challenging for both the children and their families. For example, children having to stop in the middle of a sport game to treat hypoglycemia and then wait for their blood sugar to return to a safe level before participating again, can be discouraging and frustrating [30].

For children with T1D to safely and confidently participate in PA such as recess, physical education class, or sports, a comprehensive team approach among the child, parents, coaches, and medical providers must ensue. National initiatives, grounded in research, recommend that all children, including those with health conditions, have equal opportunities to participate in sports [21,27,28,29]. Physicians and medical care teams can prescribe PA and sport participation when designing treatment plans and to refer qualified health and fitness professionals.

This study is not without limitations. The analysis was underpowered and a larger sample size may have helped reach significant findings in areas related to sport participation. The broad range in ages possibly affected the results since some of the participants had either started puberty, or were currently experiencing changes in hormone production while in the middle of puberty. Further, it seems prudent to revisit the research hypothesis with additional participants in the future. Additionally, future research should further investigate potential covariates of the relationship between sport participation, PA, and HbA1c levels that were identified in the present study and other variables not collected here. It would also be necessary to make sure that any measurements related to diabetes management (i.e., HbA1c) are collected within a similar time-frame as sporting seasons, which this study did not do. Some participants may not have been actively participating in a sport within the past 6 months or more prior to the HbA1c reported in this study and we acknowledge this had an impact on our findings.

## 5. Conclusions

HbA1c levels showed a trending decrease with each day a child engaged in PA, only the number of days a child was active per week was a significant predictor of better HbA1c levels. Clinical applications of this study center around the idea that healthcare providers should be educated about the positive influence that PA and sport participation may have on HbA1c levels in their patients living with T1D.

To develop a truly collaborative clinical and translational research effort in which research and clinical practice work in tandem to help inform each other, it is important for health professionals and researchers to understand children’s PA and sport participation characteristics and their impact on the glucose response to ensure safety. Understanding patients’ demographic characteristics, physical activity, and sport participation behaviors, and diabetes monitoring, treatment plans, and outcomes may aid sports medicine programs in developing sport-specific programs, identifying specific sports teams in which to partner, and developing sport- and PA-specific recommendations. Since the number of days active per week was a significant predictor of better HbA1c, it behooves diabetes care teams to encourage PA in addition to sport participation alone. Further investigation should address socioecological barriers to PA and sport participation.

## Figures and Tables

**Table 1:** Demographic characteristics and diabetes treatment measures for all participants and ANOVA results separated by physical activity group.

Variable	Category	All	Inactive	Active	Most Active	*p*-Value
Number		152	51 (33.55%)	50 (32.9%)	51 (33.55%)	
Age (avg. yrs)		13 ± 3	14 ± 3	13 ± 3	13 ± 3	0.12
Gender						0.25
	Female	71 (46.71%)	26 (50.98%)	26 (52%)	19 (37.25%)	
	Male	81 (53.29%)	25 (49.02%)	24 (48%)	32 (62.75%)	
Race						0.05
	Black	13 (8.55%)	8 (15.69%)	1 (2%)	4 (7.84%)	
	White	134 (88.16%)	41 (80.39%)	46 (92%)	47 (92.16%)	
	Other	5 (3.29%)	2 (3.92%)	3 (6%)	0	
Ethnicity						0.07
	Hispanic or Latino	5 (3.29%)	4 (7.84%)	1 (2%)	0	
	Not Hispanic or Latino	147 (96.71%)	47 (92.16%)	49 (98%)	51 (100%)	
						
CGM Use						0.73
	Yes	77 (50.66%)	24 (47.06%)	25 (50%)	28 (54.9%)	
	No	75 (49.34%)	27 (52.94%)	25 (50%)	23 (45.1%)	
Insulin Pump						0.85
	Yes	115 (75.66%)	40 (78.43%)	37 (74%)	38 (74.51%)	
	No	37 (24.34%)	11 (21.57%)	13 (26%)	13 (25.49%)	
Years diagnosed with T1D		4.78 ± 3.91	5.67 ± 4.06	3.84 ± 3.59	4.82 ± 3.91	0.06
Height (m)		156.54 ± 15.60	158.43 ± 14.83	154.32 ± 15.28	157.12 ± 16.67	0.40
Weight (kg)		54.36 ± 16.97	57.30 ± 18.92	53.32 ± 14.99	52.45 ± 16.67	0.31
TMI (kg/m^3^)		13.91 ± 2.85	14.09 ± 3.14	14.34 ± 2.75	13.31 ± 2.59	0.16
HbA1c		8.75 ± 1.81	9.42 ± 2.18	8.37 ± 1.70	8.46 ± 1.29	0.007
Sport Participation						<0.001
	Yes	97 (63.82%)	22 (43.14%)	35 (70%)	40 (78.43%)	
	No	55 (36.18%)	29 56.86%)	15 (30%)	11 (21.57%)	
Days of PA		3.49 ± 1.95	1.29 ± 0.83	3.48 ± 0.50	5.69 ± 0.84	<0.001
Insurance Type						
	Private Company	87 (57.24%)	29 (56.86%)	30 (60%)	28 (54.9%)	0.88
	Medicare/Medicaid	122 (80.26%)	46 (90.2%)	39 (78%)	37 (72.55%)	0.07
	None	2 (1.32%)	1 (1.96%)	0	1 (1.96%)	0.61

Abbreviations: HbA1C = glycated hemoglobin; T1D = Type 1 Diabetes; PA = physical activity; CGM = continuous glucose monitor; TMI = Tri-ponderal mass index.

**Table 2:** Adjusted associations of sociodemographic, anthropometric, and physical activity with glycated hemoglobin (*n* = 152).

Independent Variable HbA1c								
	Model 1 ^a^				Model 2 ^b^			
Variable	β (SE)	Standardized Beta	*t*	*p*-Value	β (SE)	Standardized Beta	*t*	*p*-Value
Intercept	11.14 (1.35)		8.23	<0.01	13.2 (2.32)		5.69	<0.001
Days of PA per week	−0.15 (0.07)	−0.16	−2.08	0.03	−0.17 (0.07)	−0.18	−2.26	0.02
Age	0.06 (0.05)	0.09	1.06	0.29	0.04 (0.05)	0.06	0.72	0.47
Race	−0.66 (0.29)	−0.17	−2.25	0.03	−0.73 (0.34)	−0.19	−2.12	0.04
CGM use	−0.66 (0.28)	−0.18	−2.40	0.02	−0.74 (0.28)	−0.21	−2.69	0.01
Diagnosis Duration	0.08 (0.04)	0.17	2.15	0.03	0.09 (0.04)	0.19	2.30	0.02
Gender					−0.50 (0.29)	−0.14	−1.73	0.09
Ethnicity					−0.27 (0.92)	−0.03	−0.30	0.77
TMI (kg/mt^2^)					−0.02 (0.05)	−0.03	−0.30	0.76
Insulin Pump use					0.04 (0.32)	0.01	0.13	0.89
Insurance Type					−0.70 (0.29)	−0.15	−1.94	0.06

Abbreviations: Hb A1C = glycated hemoglobin; PA = physical activity; CGM = continuous glucose monitor; TMI = Tri-ponderal mass index. ^a^ Model 1 = adjusted for physical activity, age, race, diagnosis duration, and CGM use. ^b^ Model 2 = adjusted for physical activity, age, race, CGM use, diagnosis duration in years, gender, ethnicity, TMI, insulin pump use, and insurance type.

## Data Availability

The data presented in this study are available on request from the corresponding author.
